# Synthesis of Quaternary Ammonium Salts of Chitosan Bearing Halogenated Acetate for Antifungal and Antibacterial Activities

**DOI:** 10.3390/polym10050530

**Published:** 2018-05-15

**Authors:** Jingjing Zhang, Wenqiang Tan, Fang Luan, Xiuli Yin, Fang Dong, Qing Li, Zhanyong Guo

**Affiliations:** 1 Key Laboratory of Coastal Biology and Bioresource Utilization, Yantai Institute of Coastal Zone Research, Chinese Academy of Sciences, Yantai 264003, China; jingjingzhang@yic.ac.cn (J.Z.); wqtan@yic.ac.cn (W.T.); fluan@yic.ac.cn (F.L.); fdong@yic.ac.cn (F.D.); qli@yic.ac.cn (Q.L.); 2 University of Chinese Academy of Sciences, Beijing 100049, China

**Keywords:** quaternary ammonium salts of chitosan, halogenated acetate, characterization, antifungal activity, antibacterial activity

## Abstract

In this paper, quaternary ammonium salts of chitosan bearing halogenated acetate, including *N*,*N*,*N*-trimethyl chitosan chloroacetate (TMCSC), *N*,*N*,*N*-trimethyl chitosan dichloroacetate (TMCSDC), *N*,*N*,*N*-trimethyl chitosan trichloroacetate (TMCSTC), and *N*,*N*,*N*-trimethyl chitosan trifluoroacetate (TMCSTF), were prepared via *N*,*N*,*N*-trimethyl chitosan iodide (TMCSI). The obtained chitosan derivatives were characterized by FT-IR, ^1^H NMR spectra, ^13^C NMR spectra, and elemental analysis. Their antifungal property against *Fusarium oxysporum* f. sp. cucumebrium Owen (*F. oxysporum* f. sp. cucumebrium Owen), *Botrytis cinerea* (*B. cinerea*), and *Phomopsis asparagi* (*P. asparagi*) were evaluated by hyphal measurement method at concentrations ranging from 0.08 mg/mL to 0.8 mg/mL. Meanwhile, two common bacteria, *Escherichia coli* (*E. coli*) and *Staphylococcus aureus* (*S. aureus*), were selected as the model Gram-negative and Gram-positive bacteria to evaluate the antibacterial property of the chitosan derivatives by agar well diffusion method. The results showed that TMCSC, TMCSDC, TMCSTC, and TMCSTF had better antifungal and antibacterial activities than chitosan and TMCSI. In particular, a rule showed that the inhibitory activity decreased in the order: TMCSTF > TMCSTC > TMCSDC > TMCSC > TMCSI > chitosan, which was consistent with the electron-withdrawing property of different halogenated acetate. Apparently, the quaternary ammonium salts of chitosan with stronger electron withdrawing ability possessed relatively greater antifungal and antibacterial activities. This experiment provides a potentially feasible method for the further utilization of chitosan in fields of antifungal and antibacterial biomaterials.

## 1. Introduction

Phytopathogenic fungi have been severe constraints of crop production and food security globally, which may lead to great losses to humans [1]. In addition, *E. coli* and *S. aureus* have historically been the major human pathogen and continue to be the most commonly implicated bacteria causing human disease [2]. In the past few decades, the large-scale use of chemical fungicides and antibiotics was the dominant tool to address these disasters [3]. However, the extensive application of such chemical fungicides and antibiotics often causes drug resistance in the fungi and bacteria. Drug-resistant bacterial and fungal strains can pose a serious threat to human health globally and it obliges researchers to seek new alternatives [4,5]. Therefore, developing secure and ecofriendly alternatives that can control microorganisms as well as decrease environmental risk has become an effective tactic [6].

As one of the most abundant natural polysaccharides, chitosan has unique physiochemical characteristics and bioactivities including biocompatibility, low toxicity, hemostatic activity, wound-healing properties, and antimicrobial activity that can be applied in the fields of biotechnology, pharmaceutics, wastewater, cosmetics, agriculture, food science, textiles, and so on [7,8,9,10]. Meanwhile, due to these excellent properties, chitosan can be considered as a great alternative of antimicrobial medicine or fungicide. However, the application of pristine chitosan as a fungicide has been limited due to its lower solubility and weaker activity compared to current antimicrobials on the market [11,12,13]. In order to meet the requirements of commercial medicine or fungicides, modifying the structure of chitosan is a common means to improve its solubility and other properties. Exactly, various derivatives of chitosan with better water solubility and bioactivities have been synthesized through chemical modification such as Schiff bases of chitosan, *N*-substituted chitosan, carboxymethyl chitosan, quaternized chitosan, and so on [14,15]. Among various derivatives of chitosan, quaternized chitosan has drawn people’s attention for its excellent bioactivity, especially antimicrobial and antioxidant activities. Furthermore, we found that quaternized chitosan had better antioxidant activity than chitosan, Schiff bases of chitosan, and *N*-substituted chitosan, which was caused by the cation of quaternized chitosan [14]. And the antifungal and antioxidant activities of quaternized chitosan are affected by the active groups, which grafted to chitosan according to earlier reports [12,16].

Except quaternized chitosan, chitosan ammonium salt is another kind of chitosan derivative which has an obvious cation. Meanwhile, the better antifungal activity of chitosan ammonium salt than chitosan against *C. cucumerinum* (Ell.) et Arthur, *M. fructicola* (Wint.) Honey, and *F. oxysporum* f. sp. cucumis sativus L were also reported in a past paper [17]. Now that both quaternized chitosan and chitosan ammonium salt have more positive charge, which is the principal factor that causes the enhancement of chitosan’s bioactivities, quaternary ammonium salt of chitosan may be an excellent antifungal or antibacterial agent. In addition, the electron-withdrawing substitution–halogens can play key roles in the antifungal and antibacterial properties of compounds, which can disrupt cell walls and membranes to lead to the death of microorganisms [18,19,20]. Therefore, the introduction of these groups bearing various types and quantities of halogens into quaternary ammonium salt of chitosan could help enhance the bioactivities of quaternary ammonium salt of chitosan.

In order to study the effect of halogen atoms on the bioactivities of chitosan, the synthesis of TMCSC, TMCSDC, TMCSTC, and TMCSTF were reported. Firstly, the C_2_–NH_2_ of chitosan was modified as quaternized chitosan, which acted as an intermediate that could form quaternary ammonium salts of chitosan bearing different halogenated acetate. Then, the final products were synthesized through ion exchange between *N*,*N*,*N*-trimethyl chitosan iodide and sodium haloacetic. The chemical structures of the derivatives were characterized by FT-IR, ^1^H NMR spectra, ^13^C NMR spectra, and elemental analysis. Meanwhile, their antifungal activity against *Fusarium oxysporum* f. sp. cucumerium Owen (*F. oxysporum* f. sp. cucumerium Owen: ATCC42357), *Botrytis cinerea* (*B. cinerea*: ATCC48340), and *Phomopsis asparagi* (*P. asparagi*: ATCC24625) were measured in this paper. Furthermore, we tested the inhibiting ability of synthesized derivatives with bacteria, including *Escherichia coli* (*E. coli*: ATCC27325) and *Staphylococcus aureus* (*S. aurous*: ATCC25923). Accordingly, we have discussed the relationship between the structures and bioactivities of chitosan derivatives briefly.

## 2. Materials and Methods

### 2.1. Materials

Chitosan was purchased from Qingdao Yunzhou Biochemistry Co., Ltd. (Qingdao, China). The degree of deacetylation was 81% and the viscosity average molecular weight was 7.8 × 10^3^. Iodomethane, sodium iodide, chloroacetic acid, dichloroacetic acid, trichloroacetic acid, and trifluoroacetic acid were purchased from Sigma-Aldrich Chemical Corp. (Shanghai, China). *N*-methyl-2-pyrrolidone, ethanol, sodium hydroxide, and sodium chloride were all purchased from Sinopharm Chemical Reagent Co., Ltd. (Shanghai, China). The reagents were analytical grade and used without further purification. Fungi Medium was purchased from Qingdao Hop Bio-Technogy Co., Ltd. (Qingdao, China). Agar Powder was purchased from Beijing Chembase Technology Co., Ltd. (Beijing, China). Tryptone and Yeast Extract Powder were purchased from Beijing Aoboxing Bio-Tech Co., Ltd. (Beijing, China).

### 2.2. Analytical Methods

#### 2.2.1. Fourier Transform Infrared (FT-IR) Spectroscopy

FT-IR spectrometers, in the range of 4000–400 cm^−1^ with resolution of 4.0 cm^−1^, were recorded on a Jasco-4100 (Tokyo, Japan, provided by JASCO China (Shanghai) Co. Ltd., Shanghai, China). All samples were ground and mixed with KBr disks for testing.

#### 2.2.2. Nuclear Magnetic Resonance (NMR) Spectroscopy

^1^H Nuclear Magnetic Resonance (^1^H NMR) spectra and ^13^C Nuclear Magnetic Resonance (^13^C NMR) spectra were all measured using a Bruker AVIII-500 Spectrometer (500 MHz, Switzerland, provided by Bruker Tech. and Serv. Co., Ltd., Beijing, China) at 25 °C and using 99.9% Deuterium Oxide (D_2_O) as solvents.

#### 2.2.3. Elemental Analysis

The elemental analyses by combustion were used to evaluate the degrees of substitution in chitosan derivatives. The analyses of elemental carbon, hydrogen, and nitrogen in chitosan derivatives were performed on a Vario Micro Elemental Analyzer (Elementar, Germany). The degrees of substitution (DS) of chitosan derivatives were calculated on the basis of the percentages of carbon and nitrogen according to the following equations [21]:

$$DS_{1}=\frac{n_{1}\times M_{C}-M_{N}\times \;W_{C/N}}{n_{2}\times M_{C}}$$

$$DS_{2}=\frac{M_{N}\times \;W_{C/N}+n_{2}\times M_{C}\times DS_{1}-n_{1}\times M_{C}}{n_{3}\times M_{C}}$$

$$DS_{3}=\frac{M_{N}\times \;W_{C/N}+n_{2}\times M_{C}\times DS_{1}-n_{1}\times M_{C}-n_{3}\times M_{C}\times DS_{2}}{n_{4}\times M_{C}}$$

where *DS*_1_, *DS*_2_, and *DS*_3_ represent the deacetylation degree of chitosan, the degrees of substitution of *N*,*N*,*N*-trimethyl chitosan iodide, and degrees of substitution of quaternary ammonium salts of chitosanbearing halogenated acetate, respectively; *M_C_* and *M_N_* are the molar masses of carbon and nitrogen, *M_C_* = 12, *M_N_* = 14, respectively; *n*_1_, *n*_2_, *n*_3_, and *n*_4_ are the number of carbons of chitin, acetamido group, trimethyl, and haloacetic acid group, *n*_1_ = 8, *n*_2_ = 2, *n*_3_ = 3, *n*_4_ = 2, respectively; *W_C/N_* represents the mass ratio between carbon and nitrogen in chitosan derivatives.

### 2.3. Synthesis of Chitosan Derivatives

#### 2.3.1. Synthesis of *N,N,N*-Trimethyl Chitosan Iodide (TMCSI)

TMCSI was prepared according to the following method [1]: Chitosan (10 mmol) was dispersed in 80 mL of *N*-methyl-2-pyrrolidone (NMP) and stirred at room temperature for 1 h. Then, NaI (4.5 g), 15% NaOH aqueous solution (15 mL), and CH_3_I (15 mL) were added, subsequently. The mixture was refluxed for an additional 2 h at 60 °C. After reflux reaction, the solution was poured into ethanol to afford some flavescent precipitate and TMCSI collected by filtration was obtained by freeze-drying overnight in vacuum.

#### 2.3.2. Synthesis of Quaternary Ammonium Salt of Chitosan Bearing Halogenated Acetate

**Scheme 1:**
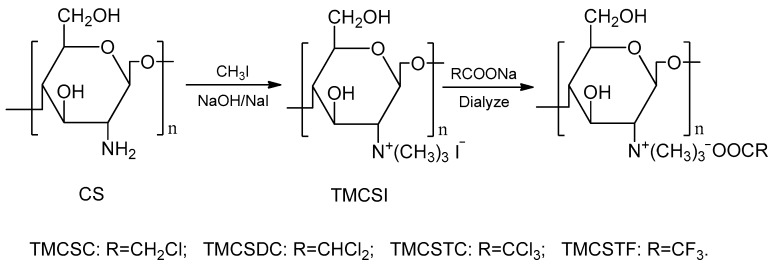
Synthesis pathway of chitosan derivatives.

TMCSI was then dissolved in 15% of sodium haloacetic in order to replace the iodide ions with haloacetic ions. The solution was dialyzed with distilled water for 3 days, and quaternary ammonium salts of chitosan bearing halogenated acetate including *N*,*N*,*N*-trimethyl chitosan chloroacetate (TMCSC), *N*,*N*,*N*-trimethyl chitosan dichloroacetate (TMCSDC), *N*,*N*,*N*-trimethyl chitosan trichloroacetate (TMCSTC), and *N*,*N*,*N*-trimethyl chitosan trifluoroacetate (TMCSTF) were obtained after being freeze-dried (Scheme 1).

### 2.4. Antifungal Assays

The antifungal ability was assessed by the model of the earlier method [22]. Firstly, the samples (chitosan and chitosan derivatives) were dispersed in distilled water at a concentration of 6 mg/mL as stock solutions. Then, each sample solution was added to fungi media, which was prepared from a mixture with a mass ratio of fungi medium, agar powder, and sterile water of 2.85:1.8:100, respectively, to give final concentrations of 0.08, 0.4, and 0.8 mg/mL. The final solutions were poured into sterilized Petri dishes (9 cm). After the mixture was cooled, the fungi mycelium of 5.0 mm diameter was transferred to the test plate and incubated at 27 °C for 2–3 days. When the mycelium of fungi reached the edges of the control plate (without added samples), the antifungal index was calculated as follows:

Antifungal index (%)=(1−DaDb) ×100

where D_a_ is the diameter of the growth zone in the test plates and D_b_ is the diameter of the growth zone in the control plate.

### 2.5. Antibacterial Assays

The antibacterial ability of chitosan and chitosan derivatives was determined using the agar well diffusion method [23,24]. Firstly, autoclaved (sterile) nutrient agar media (a mixture of Tryptone, yeast extract powder, agar powder, sodium chloride, and sterile water in the mass ratio of 10:5:18:10:1000) was prepared. Then, it was poured into sterile plates and cultured media were allowed to solidify. 0.1 mL *E. coli* and *S. aureus* (10^5^ CFU/mL) were inoculated on the nutrient agar media and then spread on the entire surface of the medium by sterile spatula. Next, four wells of 5 mm diameter were bored using a sterile borer in each plate. In each well, 25 μL of samples (at the concentration of 20 mg/mL) were loaded. Bacterial plates were incubated at 37 °C overnight and zones of inhibition were measured after 24 h. Greater inhibition zones indicated higher antibacterial activity.

### 2.6. Statistical Analysis

All the experiments were performed in triplicate and the data were expressed as means ± the standard deviation (SD, *n* = 3). Significant difference analysis was determined using Scheffe’s multiple range test. A level of *p* < 0.05 was considered statistically significant.

## 3. Results and Discussion

### 3.1. Structure of Quaternary Ammonium Salts of Chitosan Bearing Halogenated Acetate

**Figure 1:**
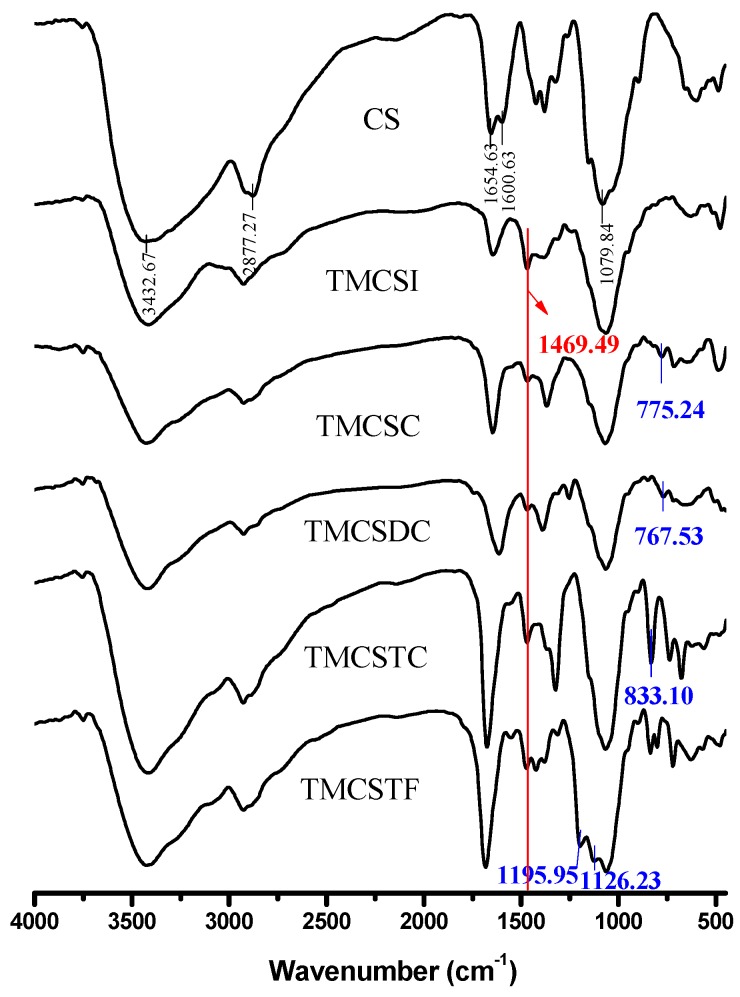
FT-IR spectra of chitosan, TMCSI, TMCSC, TMCSDC, TMCSTC, and TMCSTF.

**Figure 2:**
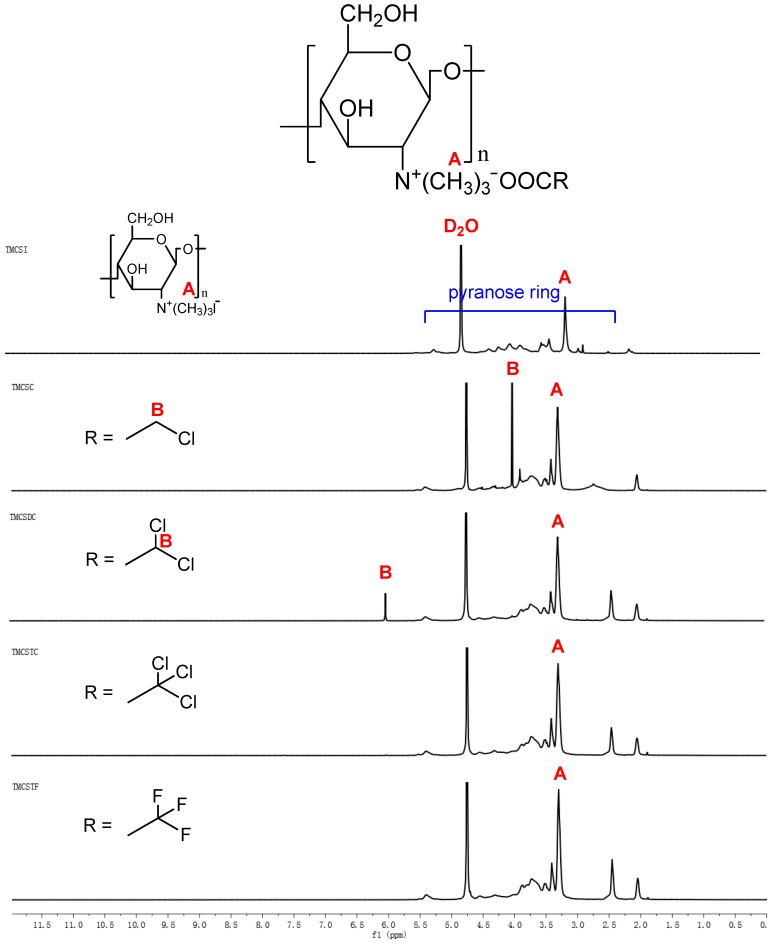
^1^H NMR spectra of TMCSI, TMCSC, TMCSDC, TMCSTC, and TMCSTF.

**Figure 3:**
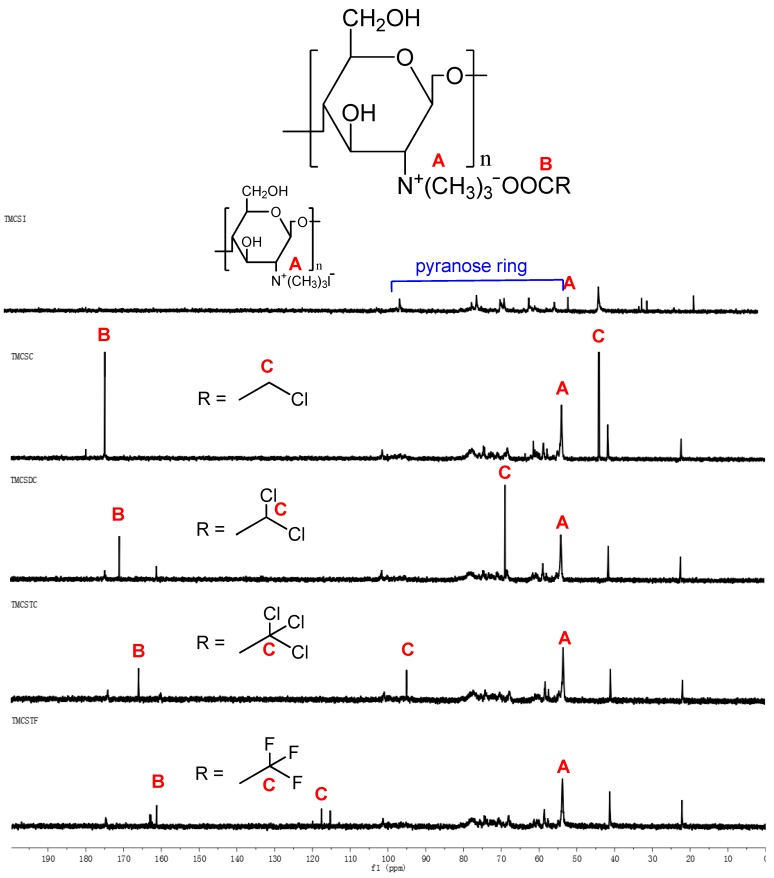
^13^C NMR spectra of TMCSI, TMCSC, TMCSDC, TMCSTC, and TMCSTF.

Each step of the synthesis was followed by FT-IR spectra (Figure 1), ^1^H NMR spectra (Figure 2), and ^13^C NMR spectra (Figure 3). Meanwhile, the elemental analyses, yields, and the degrees of substitution of quaternary ammonium salts of chitosan bearing halogenated acetate are shown in Table 1.

The FT-IR spectra of chitosan, TMCSI, TMCSC, TMCSDC, TMCSTC, and TMCSTF are shown in Figure 1. The spectrum of chitosan shows characteristic absorption bands at approximately 3432.67 cm^−^^1^ (angular deformation of O–H and N–H), 2877.27 cm^−1^ (–CH stretching), 1600.63 cm^−1^ (vibration modes of amino group), and 1079.84 cm^−1^ (the absorbance of β-(1,4) glycosidic in chitosan) [25,26,27,28]. For TMCSI, a new peak appears at about 1469 cm^−1^, which is assigned to the characteristic absorbance of N–CH_3_ [1,16]. For TMCSC, TMCSDC, and TMCSTC, there are new peaks appearing at 775.24, 767.53, and 833.10 cm^−1^, respectively, which should be assigned to the absorbance of the C–Cl bond [12]. Meanwhile, new peaks appear at 1195.95 and 1126.23 cm^−1^ for TMCSTF, which are assigned to the characteristic absorbance of C–F [12]. At the same time, there are characteristic peaks of N–CH_3_ at about 1470 cm^−1^, which prove that the quaternary ammonium salts groups still exist in the molecules of TMCSC, TMCSDC, TMCSTC, and TMCSTF. The above-mentioned results preliminarily demonstrated that quaternary ammonium salts of chitosan derivatives were obtained.

The structures of the synthesized products were further demonstrated by ^1^H NMR spectra (Figure 2) and ^13^C NMR spectra (Figure 3). As shown in the figures, all spectra show signals at 2.0–5.5 ppm (^1^H NMR spectra) and 50–100 ppm (^13^C NMR spectra), which are assigned to the diagnostic chemical shifts of chitosan [29,30]. In ^1^H NMR spectrum of TMCSI, the chemical shift at 3.1 ppm is assigned to the protons of –N^+^(CH_3_)_3_ groups [1,31]. Similarly, the trimethyl groups show the prominent peak at 53 ppm in ^13^C NMR spectrum. As compared with TMCSI, new peaks are observed at 4.0 ppm (CH_2_ClCOO– in TMCSC) and 6.0 ppm (CHCl_2_COO– in TMCSDC) in ^1^H NMR spectra of products, which are assigned to protons of halogenated acetic anions [32]. As far as TMCSTC and TMCSTF, the spectra are similar to TMCSI and no new peaks appear because of absence of protons of halogenated acetic anions. As to the ^13^C NMR spectra of quaternary ammonium salts of chitosan bearing halogenated acetate, new signals at about 175, 170, 166, and 161 ppm are related to carbons of COO– groups in TMCSC, TMCSDC, TMCSTC, and TMCSTF [33]. Furthermore, new resonance peaks appear at 44 ppm (CH_2_Cl in TMCSC), 69 ppm (CHCl_2_ in TMCSDC), 95 ppm (CCl_3_ in TMCSTC), and 117 ppm (CF_3_ in TMCTF), confirming the presence of the halogenated methyl carbons [12,34]. In addition, the chemical shifts of –N^+^(CH_3_)_3_ at about 3.1 ppm (^1^H NMR spectra) and 53 ppm (^13^C NMR spectra) still exist in the spectra of quaternary ammonium salts of chitosan bearing halogenated acetate. Therefore, these data adequately prove the successfully synthesis of chitosan derivatives.

The degrees of substitution (DS) for chitosan derivatives were calculated on the basis of the percentages of carbon and nitrogen and the results are shown in Table 1. As is shown, the intermediate TMCSI presents the highest degree of substitution. The DS of four final products (TMCSC, TMCSDC, TMCSTC, and TMCSTF) are various. For example, compared with TMCSDC with the DS of 0.60, the DS value of TMCSTF is only 0.32. Generally, the degrees of substitution of products would directly affect their bioactivity. The lower degree of substitution as well as less functional groups mean a weaker bioactivity. Therefore, even if TMCSTF has the strongest electronegativity, its biological activity may not be the best because of the low DS in some cases.

### 3.2. Antifungal Activity

**Figure 4:**
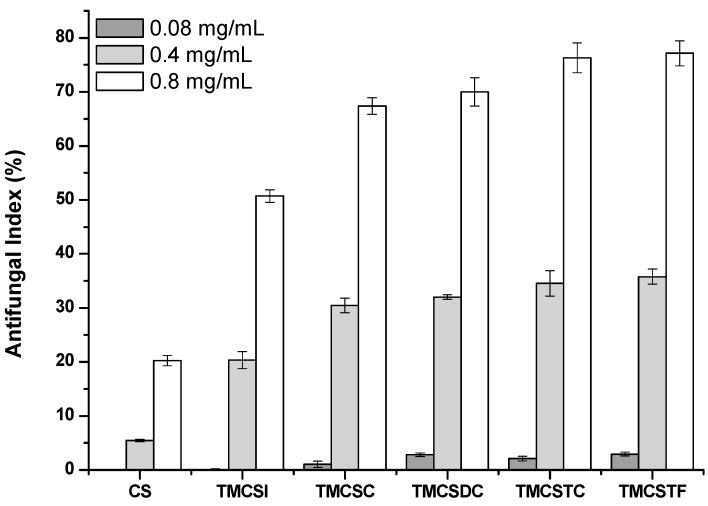
The antifungal activity of chitosan and chitosan derivatives against *F. oxysporum* f. sp. cucumebrium Owen.

**Figure 5:**
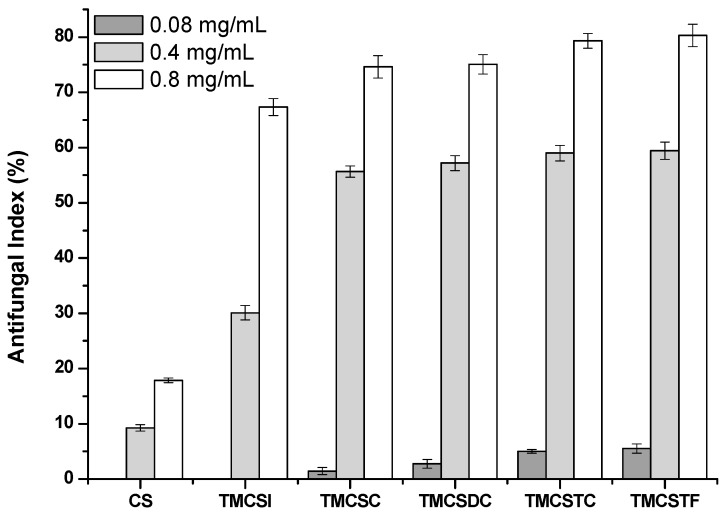
The antifungal activity of chitosan and chitosan derivatives against *B. cinerea*.

**Figure 6:**
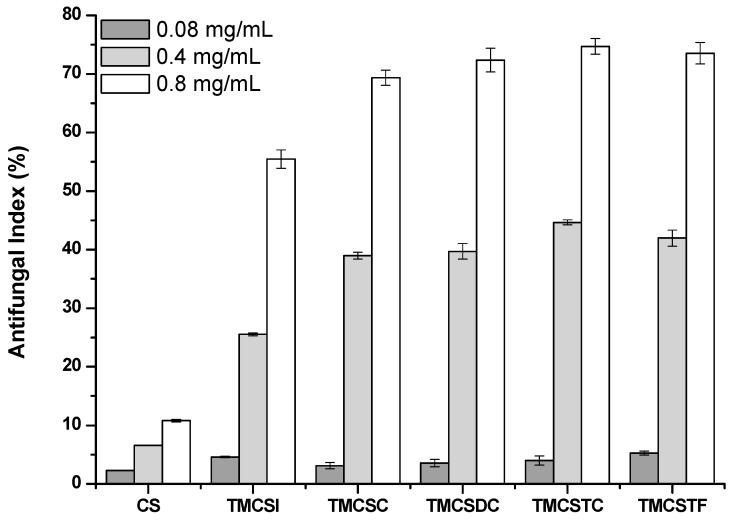
The antifungal activity of chitosan and chitosan derivatives against *P. asparagi*.

The antifungal activity of chitosan and chitosan derivatives against three destructive phytopathogenic fungi, including *F. oxysporum* f. sp. cucumebrium Owen, *B. cinerea*, and *P. asparagi*, are shown in Figure 4, Figure 5 and Figure 6. Their antifungal indices and the relationship between the structure and antifungal activity of chitosan derivatives are discussed as follows.

*F. oxysporum* is one of the most important soil-borne pathogens that cause root-rot and wilt diseases in a wide variety of crops. It has the ability to persist for very long periods in soil without a host [35]. Figure 4 shows the antifungal activity of chitosan and all derivatives against *F. oxysporum* f. sp. cucumebrium Owen. As is shown, several conclusions can be gained as follows: firstly, for all tested samples, the inhibitory rates increase with the rise in concentration. This trend is obvious and is suitable for all samples. For instance, the inhibitory rates of TMCSC are 1.01%, 30.47%, and 67.38% when the corresponding concentrations are 0.08 mg/mL, 0.4 mg/mL, and 0.8 mg/mL, respectively. Secondly, because of the introduction of electron-drawing groups–halogenated acetate, the final products have a better ability to inhibit *F. oxysporum* f. sp. cucumebrium Owen compared with chitosan and TMCSI, and this conclusion is apparently reinforced at 0.8 mg/mL and 0.4 mg/mL. Thirdly, of all the tested samples, the antifungal activity against *F. oxysporum* f. sp. cucumebrium Owen decreases in the order: TMCSTF > TMCSTC > TMCSDC > TMCSC > TMCSI > chitosan. This regularity is identical with the sort order of electronegativity (–CF_3_ > –CCl_3_ > –CHCl_2_ > –CH_2_Cl) of substituent groups with halogens in chitosan quaternary ammonium salts. For example, the antifungal indices of TMCSTF, TMCSTC, TMCSDC, TMCSC, TMCSI, and chitosan are 77.15%, 76.30%, 70.01%, 67.38%, 50.72%, and 20.25% at 0.8 mg/mL, respectively. The results above demonstrate that the difference of active groups grafting onto the synthesized chitosan derivatives contributed much to the antifungal action, but their effect on promoting antifungal activity varied according to the difference in electronegativity.

*B. cinerea* is an airborne plant pathogen with a necrotrophic lifestyle attacking over 200 crop hosts (including field and row crops, fruit, vegetables, and flowers) and it can cause destructive and economically important plant diseases worldwide. The antifungal activity of chitosan and chitosan derivatives against *B. cinerea* was tested and the results are shown in Figure 5. Overall, most rules against *F. oxysporum* f. sp. cucumerium Owen discussed above still can be appropriate for the antifungal activity of samples against *B. cinerea.* For example, all samples exhibit antifungal properties against *B. cinerea* and the inhibitory indices are concentration-dependent. Besides, the sample that possesses the weakest inhibitory activity is still chitosan. The quaternary ammonium salts of chitosan bearing halogenated acetate (TMCSC, TMCSDC, TMCSTC, and TMCSTF) also have better antifungal ability than TMCSI and chitosan. Furthermore, the rule of inhibitory property against *B. cinerea*, which is TMCSTF > TMCSTC > TMCSDC > TMCSC > TMCSI > chitosan, is distinct according to Figure 5. The inhibitory indices of TMCSTF, TMCSTC, TMCSDC, TMCSC, TMCSI, and chitosan at 0.4 mg/mL against *B. cinerea* are 59.43%, 59.02%, 57.2%, 55.66%, 30.10%, and 9.25%, respectively.

As a destructive pathogenic fungus, *P. asparagi* can cause severe stem blight of asparagus. Figure 6 shows the antifungal activity of chitosan and chitosan derivatives against *P. asparagi*. The inhibitory indices of all the samples mounted up with increasing concentration. All chitosan derivatives bearing halogenated acetate show better antifungal activity than TMCSI and chitosan, and the inhibitory indices of TMCSTF, TMCSTC, TMCSDC, and TMCSC are 73.54%, 74.69%, 72.36%, and 69.35% at 0.8 mg/mL, respectively, while the inhibitory rates of TMCSI and chitosan are 55.47% and 10.8%. However, the rule of inhibitory activity against *P. asparagi* is a little different from the fungi of *F. oxysporum* f. sp. cucumerium Owen and *B. cinerea*. The order, which is TMCSTC > TMCSDC > TMCSC, is in accord with earlier descriptions. The inhibitory property of TMCSTF is slightly lower than TMCSTC. Although the electronegativity of the fluorine atom is stronger than that of the chlorine atom, it is possible that the antifungal activity of TMCSTF is weaker than that of TMCSTC because of its lower substitution degree (Table 1) and less active ingredient. In brief, these results mentioned above indicate that the halogen atoms have a positive effect on antifungal activity when they are introduced into chitosan and the mechanism between the structure and antifungal activity of chitosan derivatives will be simply discussed later.

### 3.3. Antibacterial Activity

The antibacterial activity against *E. coli* and *S. aureus* of chitosan and chitosan derivatives were evaluated through the agar well diffusion method. The results depicted in Table 2 reveal that the tested samples display a moderate inhibitory effect on the growth of bacterial pathogens. In general, the results drawn from antibacterial activity are similar to antifungal activity. For instance, all of the chitosan derivatives exhibit better antibacterial activity comparable to chitosan. Moreover, the antibacterial activity decreases in the order of TMCSTF > TMCSTC > TMCSDC > TMCSC > TMCSI > chitosan, which is identified with antifungal activity. The above results therefore suggest that different substituents can markedly influence the antibacterial efficacy of the compounds and the stronger electron-withdrawing capacity indicates better antibacterial activity.

According to earlier reports, chitosan and its derivatives possess broad spectrum antimicrobial activity and this activity can be affected by several factors such as pH, concentration, molecular weight, degree of deacetylation, temperature, and so on with different mechanisms of action [36,37,38]. In this paper, the halogenated acetate that was selected to be grafted on chitosan is another important factor that could influence the bioactivity of chitosan. Up to now, many researches have proven that the introduction of halogen atoms into the polysaccharide derivatives causes an increase in biological activity. For example, Xie et al. had reported that grafting coumarin groups bearing different halogen atoms on chitosan obviously enhanced the antifungal activity of the synthesized chitosan derivatives compared with unmodified chitosan [3]. Furthermore, the starch derivatives containing halogenated benzene showing higher antibacterial activity than unmodified starch was demonstrated by Guo et al. [39]. They also reported that halogens could improve the biological activity of derivatives by increasing the distribution of electron clouds in compounds. In this study, the direct enhancement of antifungal and antibacterial activities of halogenated acetate grafting onto chitosan derivatives can be confirmed by the data mentioned above. Meanwhile, this conclusion is further confirmed by the active rule, which is TMCSTF > TMCSTC > TMCSDC > TMCSC > TMCSI > chitosan. Apparently, the rule is consistent with the electron-withdrawing property of different halogenated acetate and the reasons can be analyzed from the following three aspects: firstly, because the stronger electron-withdrawing capacity of halogen atoms has a positive effect on the bioactivity, the inhibitory properties of TMCSTF, TMCSTC, TMCSDC, and TMCSC are higher than TMCSI and chitosan. Exactly, the halogen atoms with high electron-withdrawing property could increase the hydrophobicity at the periphery of the synthesized chitosan derivatives, and it can also increase the intermolecular and intramolecular delocalization of the electronic cloud distribution in the synthesized compounds. These effects have an impact in leading to the significant alteration of the structure of the outer membranes as well as the internal membranes of microbial cells, which hinders nutrient substances from entering cells and causes the release of a large proportion of proteinaceous materials from the cells. These results directly lead to the death of microorganisms [40,41,42]. Therefore, the chitosan derivatives bearing stronger electronegative groups show stronger antifungal and antibacterial activities. Then, the biological activity of chitosan derivatives varies with the types and quantities of halogen atoms. For example, TMCSTC, TMCSDC, and TMCSC bear the same types of halogen-Cl, but the capacity of electron-withdrawing increases with the quantities. Hence, the bioactivity of the product shows the order of TMCSTC > TMCSDC > TMCSC. In addition, TMCSTF and TMCSTC have the same amount of halogens, but because the electronegativity of fluorine atoms are higher than chlorine atoms, TMCSTF possesses better biological activity than TMCSTC. Of course, the electron-withdrawing property is the main factor affecting the antimicrobial law and other factors may contribute a lot to it. For instance, this rule will also be affected by the substitution degree and the special circumstance (the antifungal activity of samples against *P. asparagi*) has been discussed before. In summary, the key roles of halogenated acetate in improving the antifungal and antibacterial activities of samples and the structure–activity relationship will be investigated in the future.

## 4. Conclusions

Chitosan derivatives with positive charge have excellent biological activity, and the typical derivatives of chitosan with positive charge are quaternized chitosan and chitosan ammonium salt. In this paper, we introduced different negative ions with halogen atoms to *N*,*N*,*N*-trimethyl chitosan to give a kind of quaternary ammonium salt of chitosan, which is a novel positively charged chitosan derivative. The synthesized compounds were evaluated for their expected antibacterial and antifungal activities, where the majority of these compounds showed potent antibacterial and antifungal activities against the tested strains of bacteria and fungi. Generally, the bioactivities were mainly influenced by the electron-withdrawing capacity of the substituted groups and the quaternary ammonium salts of chitosan derivatives with stronger electronegativity groups had stronger antifungal and antibacterial activities. As we know, there are various negative ions which have strong electronegativity, and many quaternary ammonium salts of chitosan with different negative ions can be prepared in future.

## Figures and Tables

**Table 1:** The elemental analyses, yields, and the degrees of substitution of chitosan derivative.

Compounds	Yields (%)	Elemental Analyses (%)				Degrees of Substitution	Deacetylation
		C	N	H	C/N		
CS		40.851	7.474	6.879	5.47	-	0.81
TMCSI	87.6	29.211	4.076	5.485	7.17	0.66	-
TMCSC	73.5	39.28	4.941	6.461	7.95	0.46	-
TMCSDC	69.8	38.439	4.689	7.217	8.2	0.6	-
TMCSTC	75.3	43.054	5.361	7.837	8.03	0.5	-
TMCSTF	74.6	42.88	5.556	6.998	7.72	0.32	-

**Table 2:** The measurements of antibacterial activity for chitosan and chitosan derivatives by agar well diffusion method.

Microorganism	Zone of Inhibition in mm (Mean ± SD) *n* = 3					
	CS	TMCSI	TMCSC	TMCSDC	TMCSTC	TMCSTF
***E. coli***	5.11 ± 0.25	5.72 ± 0.35	7.14 ± 0.46	7.66 ± 0.52	7.77 ± 0.47	7.86 ± 0.39
***S. aureus***	5.19 ± 0.21	6.42 ± 0.53	6.62 ± 0.48	7.36 ± 0.60	8.23 ± 0.49	8.77 ± 0.41

Values are expressed as mean ± SD. Different letters within a row mark significantly different values (*p* < 0.05).
